# Knowledge, attitudes and awareness of the human papillomavirus among health professionals in New Zealand

**DOI:** 10.1371/journal.pone.0197648

**Published:** 2018-12-31

**Authors:** Susan M. Sherman, Karen Bartholomew, Hayley J. Denison, Hersha Patel, Esther L. Moss, Jeroen Douwes, Collette Bromhead

**Affiliations:** 1 School of Psychology, Keele University, Keele, Staffs, United Kingdom; 2 Waitemata District Health Board (DHB) and Auckland DHB, Auckland, New Zealand; 3 Centre for Public Health Research, Massey University, Wellington, New Zealand; 4 Department of Gynaecology, University Hospitals Leicester, Leicester, United Kingdom; 5 Leicester Cancer Research Centre, University of Leicester, United Kingdom; 6 Massey University, School of Health Sciences, Wellington, New Zealand; Public Health England, UNITED KINGDOM

## Abstract

**Background:**

Human papillomavirus (HPV) is a common sexually transmitted infection that is implicated in 99.7% of cervical cancers and several other cancers that affect both men and women. Despite the role that HPV plays in an estimated 5% of all cancers and the evolving role of HPV vaccination and testing in protecting the public against these cancers, preliminary research in New Zealand health professionals suggest knowledge about HPV may not be sufficient.

**Methods:**

A total of 230 practice nurses, smear takers and other clinical and laboratory staff who attended a range of training events completed a cross-sectional survey between April 2016 and July 2017. The survey explored four broad areas: demographics and level of experience, HPV knowledge (general HPV knowledge, HPV triage and test of cure (TOC) knowledge and HPV vaccine knowledge), attitudes towards the HPV vaccine and self-perceived adequacy of HPV knowledge.

**Results:**

The mean score on the general HPV knowledge questions was 13.2 out of 15, with only 25.2% of respondents scoring 100%. In response to an additional question, 12.7% thought (or were unsure) that HPV causes HIV/AIDS. The mean score on the HPV Triage and TOC knowledge questions was 7.4 out of 10, with only 9.1% scoring 100%. The mean score on the HPV vaccine knowledge questions was 6.0 out of 7 and 44.3% scored 100%. Only 63.7% of respondents agreed or strongly agreed that they were adequately informed about HPV, although 73.3% agreed or strongly agreed that they could confidently answer HPV-related questions asked by patients. Multivariate analyses revealed that knowledge in each domain predicted confidence in responding to patient questions. Furthermore, the number of years since training predicted both HPV knowledge and Triage and TOC knowledge.

**Discussion:**

Although overall level of knowledge was adequate, there were significant gaps in knowledge, particularly about the role of HPV testing in the New Zealand National Cervical Screening Programme. More education is required to ensure that misinformation and stigma do not inadvertently result from interactions between health professionals and the public.

## Introduction

Human papillomavirus (HPV) is responsible for 99.7% of cases of cervical cancer along with some head and neck, penile and anal cancers. There are approximately 150 new diagnoses and 50 deaths from cervical cancer in New Zealand (NZ) every year [1], while head and neck cancers attributable to HPV are increasing in both men and women with 94 new cases and 43 deaths estimated for 2012 [2]. In addition, there are longstanding ethnic inequalities in cervical cancer incidence and mortality, and cervical screening coverage remains low (and cancer incidence and mortality high) for indigenous Māori women as well as Pacific women [3].

The NZ National Cervical Screening Programme (NCSP), which was established in 1990^4^, recommends 3-yearly routine screening with liquid-based cytology (LBC) for 20–69 year-old women, with HPV triage testing for low grade (ASC-US/LSIL) cytology in women 30+ years. The programme also recommends testing of cure following treatment for a high-grade lesion [4]. From late 2018 the NCSP will introduce HPV testing as the primary screening test for women aged 25–68 years on a 5 yearly basis [5].

To reduce infection with high-risk types of HPV and its related cancers, the NZ National HPV Immunisation Programme was introduced in September 2008, offering free HPV vaccination (Gardasil, Merck) for females born in 1990 or later. School-based immunisation for 12–13 year-old girls commenced in most regions in 2009 [1] and the three-dose coverage achieved by the program in cohorts born in 1991–2002 reached approximately 48–66% nationwide [1]. In January 2017, the free programme was extended to boys and young men, the upper age for free vaccination was increased to 26 years, a two-dose schedule was implemented for individuals aged 14 and under, and the vaccine used was changed to nonavalent Gardasil 9 (Merck) [1].

Previous research has identified that health professionals can play an important role in vaccine uptake. In an Italian survey assessing childhood vaccine hesitancy in parents, hesitancy was significantly more common in those parents who lacked confidence in their child’s doctor [6]. In a US study, more adolescents had not had the HPV vaccine when their parents felt they were not able to openly discuss their concerns with the doctor [7] and in a second US study of parents who decline then later accept the HPV vaccination for their child, secondary acceptance was more likely in parents who received follow-up counselling from their child’s healthcare provider [8]. Furthermore, recent research in the UK has also identified that women who report greater trust in their doctor were less likely to have decided not to undergo cervical screening [9].

In NZ, a cervical sample taker is a registered health practitioner (nurse or doctor) who holds a current practising certificate and has completed appropriate cervical screening training as part of a medical degree, midwifery training programme or via a New Zealand Qualifications Authority (NZQA) accredited course for cervical sample takers. Previous research exploring the knowledge of GPs and practice nurses (PNs) in Christchurch, New Zealand about HPV used 5 questions as part of a larger survey exploring attitudes towards HPV vaccination [10]. Whilst performance across the 5 questions was reasonable, there was uncertainty as indicated by the number of ‘not sure’ responses, as well as some variability across questions. For example, while more than 90% of GPs and PNs knew that HPV vaccination would not eliminate the need for cervical screening, only 33% of GPs and 7% of PNs knew that anogenital warts caused by HPV 6 and 11 are not a precursor to cervical cancer. Only half of GPs and 42% of PNs knew that most HPV infections will clear without medical treatment and a quarter of GPs and nearly a third of PNs did not know, or were unsure, whether persistent HPV was a necessary cause of cervical cancer.

To our knowledge there are no studies exploring what primary care staff such as GPs, PNs and smear takers in NZ know about HPV since 2009. In light of the recent changes to the immunisation programme and the forthcoming changes to the NCSP, it is important to benchmark what nurses and smear takers understand about HPV, whether they feel well informed and assess any training needs they might identify.

## Methods

Ethics approval was granted by the Massey University Ethics Committee 4000015595. The project was registered with Waitemata DHB localities (Reference number RM13518). Both Waitemata and Auckland DHB confirmed that locality authorisation was not required as the research was carried out in community healthcare settings.

An anonymous cross-sectional survey was conducted between April 2016 and July 2017. GPs, practice nurses, smear takers and other clinical and laboratory staff who attended a variety of training events (11 in total) in Auckland District Health Board (DHB), Hutt Valley DHB and Waitemata DHB catchment areas were invited to complete the paper-based survey. The sample represents the number of respondents we were able to collect within the one-year time frame. Participants were provided with an information sheet to read prior to completing the survey. The survey was taken from Patel et al., [11] who had incorporated most of the items from Waller et al., [12] and was adapted by adding back in a question about HPV and HIV/AIDS from Waller et al., and by changing some wording to make the terminology or protocols New Zealand-specific.

We established the face validity of the adapted questionnaire for the NZ clinical environment by having two groups peer review the survey, firstly to ensure we had captured the scope adequately and secondly to ensure questions were well structured. These groups included members of the DHB Immunisation team and cervical screening specialist doctors as well as nurse practitioners.

The final survey explored four broad categories: demographics and level of experience; HPV knowledge (general HPV knowledge, HPV triage and test of cure (TOC) knowledge and HPV vaccine knowledge), which were assessed using a true, false, don’t know format; and attitudes towards the HPV vaccine and self-perceived adequacy of HPV knowledge, which were assessed using 5-point Likert scales (the survey is publicly available here: osf.io/ub7g2, DOI 10.17605/OSF.IO/UB7G2).

### Statistical analyses

Demographic factors included age, profession and years since HPV training. For analyses, profession was collapsed into four categories (nurse; general practitioner (GP); colposcopy, which included colposcopists and colposcopy nurses; and laboratory staff and other), and years since HPV training was collapsed into 3 categories (never; ≤ 1 year; > 1 year).

Factors affecting HPV knowledge were assessed using ordinal regression analysis. The approach for model development was to conduct univariate analyses initially and then enter variables into the full multivariate models that showed a statistically significant (P<0.05) association with the main outcomes in the univariate analyses. The rationale for this was that if a variable was associated with the main outcome measure, it could be a confounder.

Factors affecting self-perceived adequacy of HPV knowledge were also assessed. Feeling adequately informed and feeling confident in answering patient questions were converted from 5-point Likert scales to binary variables of yes (strongly agree, agree) and no or undecided (strongly disagree, disagree or undecided) as the dependent variables.

## Results

A total of 234 health professionals completed the survey. Due to the opportunistic nature of participant recruitment, a response rate was not able to be calculated. The data for four individuals were removed, as there were large sections that had been left unanswered. A total of 22 health professionals had at least one answer missing for the general HPV knowledge questions, 18 had at least one answer missing for the Triage and TOC knowledge questions, and 8 health professionals had at least one answer missing for the vaccine knowledge questions. Overall, 40 participants had at least one answer missing across all of the questions (several participants had missing data in more than one of the three sections). Details about participant gender, age categories, profession, smear taker status and date of most recent training, if any, are presented in Table 1.

**Table 1:** Participant characteristics.

**Gender**	**Gender**	**N (%)**
	Female	212 (92.2)
	Male	17 (7.4)
	Not specified	1 (0.4)
	Other	0 (0.0)
**Age**	**Age bracket**	**N (%)**
	20–24	8 (3.5)
	25–35	45 (19.7)
	36–45	51 (22.3)
	46–55	63 (27.5)
	56–65	52 (22.7)
	66–75	10 (4.4)
	Blank^1^	1
**Profession**	**Profession**	**N (%)**
	Registered nurse	150 (65.2)
	Laboratory staff	25 (10.9)
	General practitioner	12 (5.2)
	Not disclosed	11 (4.8)
	Colposcopist	2 (0.9)
	Colposcopy nurse	2 (0.9)
	Enrolled nurse	2 (0.9)
	Other	26 (11.3)
**Smear taking**	**Smear taker status**	**N (%)**
	Have taken a smear test^2^	123 (53.5)
	Never taken a smear test	107 (46.5)
**HPV training**	**Date of last training**	**N (%)**
	Never	99 (47.1)
	last 6 months	16 (7.6)
	7–12 months	30 (14.3)
	13–24 months	35 (16.7)
	>2yrs	30 (14.3)
	Blank^1^	20

^1^Omitted from % calculations

^2^ For the 123 who had, the years of experience ranged from 0.1 to 42 years (mean 8.7 years, median 6.5 years).

### General HPV knowledge

Out of a maximum knowledge score of 15 (see individual questions in Table 2 and excluding the question about HIV/AIDS), the mean score achieved by participants was 13.3 (standard deviation (SD) 2.0) and the median score was 14 (range 0–15, interquartile range (IQR) 13–15), with 27.9% (N = 58) achieving 100%. One individual did not answer any questions correctly.

**Table 2:** HPV and vaccine knowledge questions.

	Correct Response N (%)	Incorrect Response N (%)	Answer “don’t know” N (%)	Missing N^1^
**General HPV knowledge questions**				
HPV can cause cervical cancer (TRUE)	228 (99.1)	1 (0.4)	1 (0.4)	0
Having many sexual partners increases the risk of getting HPV (TRUE)	223 (97.0)	5 (2.2)	2 (0.9)	0
HPV can be passed on during sexual intercourse (TRUE)	218 (96.0)	2 (0.9)	7 (3.1)	3
A person could have HPV for many years without knowing it (TRUE)	217 (96.9)	0 (0)	7 (3.1)	6
HPV always has visible signs or symptoms (FALSE)	216 (94.7)	5 (2.2)	7 (3.1)	2
HPV is very rare (FALSE)	215 (93.9)	7 (3.1)	7 (3.1)	1
There are many types of HPV (TRUE)	214 (93.0)	4 (1.7)	12 (5.2)	0
Men cannot get HPV (FALSE)	213 (92.6)	8 (3.5)	9 (3.9)	0
Using condoms reduces the risk of getting HPV (TRUE)	204 (89.5)	17 (7.5)	7 (3.1)	2
HPV can be passed on by genital skin-to-skin contact (TRUE)	203 (89.0)	9 (3.9)	16 (7.0)	2
HPV can cause genital warts (TRUE)	203 (89.0)	13 (5.7)	12 (5.3)	2
HPV can be cured with antibiotics (FALSE)	201 (87.8)	14 (6.1)	14 (6.1)	1
HPV can cause HIV/AIDS^2^ (FALSE)	198 (87.2)	13 (5.7)	16 (7.0)	3
Most sexually active people will get HPV at some point in their lives (TRUE)	171 (75.3)	29 (12.8)	27 (11.9)	3
Having sex at an early age increases the risk of getting HPV (TRUE)	169 (73.8)	44 (19.2)	16 (7.0)	1
HPV usually doesn’t need any treatment (TRUE)	147 (64.2)	67 (29.3)	15 (6.6)	1
**HPV Triage and TOC knowledge questions**				
If a woman tests positive for HPV she will definitely get cervical cancer (FALSE)	220 (96.1)	5 (2.2)	4 (1.7)	1
An HPV test can be done at the same time as a Smear test (TRUE)	205 (89.5)	7 (3.1)	17 (7.4)	1
HPV testing is used to indicate if the HPV vaccine is needed (FALSE)	199 (87.3)	10 (4.4)	19 (7.9)	2
An HPV test can tell how long you have had an HPV infection (FALSE)	190 (83.0)	8 (3.5)	31 (13.5)	1
When you have an HPV test, you get the results the same day (FALSE)	189 (82.9)	8 (3.5)	31 (13.6)	2
If an HPV test shows that a woman does not have HPV her risk of cervical cancer is low (TRUE)	174 (76.0)	35 (15.3)	20 (8.7)	1
If cytology and high-risk HPV test are negative at 12 and 24 post treatment, they will need require a repeat smear in 3 Years^3^ (TRUE)	171 (75.7)	25 (11.1)	30 (13.3)	4
If high-risk HPV test is negative at 12 and 24 post treatment they will still require annual follow up for life^3^ (FALSE)	140 (60.9)	52 (22.6)	38 (16.5)	0
All cervical samples taken 6 to 12 months post-treatment can be tested for high-risk HPV^3^ (TRUE)	102 (45.1)	31 (13.7)	93 (41.2)	4
All cervical samples showing mild cellular (ASC-US/LSIL) are tested for high-risk HPV^3^ (FALSE)	101 (44.7)	85 (37.6)	40 (17.7)	4
**HPV vaccine knowledge questions**				
The HPV vaccines offer protection against all sexually transmitted infections (FALSE)	218 (96.0)	3 (1.3)	6 (2.6)	3
Girls who have had the HPV vaccine do not need to have smear tests when they are older (FALSE)	218 (94.8)	2 (0.9)	10 (4.3)	0
Someone who has had HPV vaccine cannot develop cervical cancer (FALSE)	205 (90.3)	8 (3.5)	14 (6.2)	3
The recommended number of HPV vaccine doses is three^3^,^4^ (TRUE)	202 (88.2)	7 (3.1)	20 (8.7)	1
The HPV vaccines are most effective if given to people who have never had sex (TRUE)	189 (82.5)	25 (10.9)	15 (6.6)	1
The HPV vaccines offer protection against most cervical cancers (TRUE)	185 (81.1)	29 (12.7)	14 (6.1)	2
The HPV vaccine offers protection against genital warts (TRUE)	156 (68.1)	41 (17.9)	32 (14.0)	1

^1^Omitted from % calculations

^2^Question from Waller et al [12] and in addition to Patel et al [11]

^3^Wording altered from Patel et al [11] for New Zealand context

^4^A two-dose schedule for individuals aged 14 and under was implemented in January 2017 so it is possible that some of the No/Don’t know responses reflected this fact.

The following questions were most often answered incorrectly: HPV usually doesn’t need any treatment (35.9% answered incorrectly or weren’t sure); Having sex at an early age increases the risk of getting HPV (26.2%); Most sexually active people will get HPV at some point in their lives (24.7%). In addition, more than 10% of health professionals incorrectly thought (or were not sure) that HPV cannot be passed on by genital skin-to-skin contact, that HPV does not cause genital warts, that using condoms does not reduce the risk of getting HPV and that HPV can be cured with antibiotics.

Following Waller et al., [12] the item about HIV/AIDS was analysed separately from the rest of the questions. In total, 87.2% of respondents correctly identified that HPV does not cause HIV/AIDS.

### HPV Triage and TOC knowledge

Out of a maximum knowledge score of 10 (see individual questions in Table 2), the mean score achieved by the participants was 7.4 (SD 2.0) and the median score was 8 (range 0–10, IQR 6–9), with 9.9% (N = 21) achieving 100%. Three individuals had no correct answers.

The following questions were answered incorrectly most often: All cervical samples showing mild cellular (ASC-US/LSIL) are tested for high-risk HPV (55.3% answered incorrectly or were not sure); All cervical samples taken 6 to 12 months post-treatment can be tested for high-risk HPV (54.9%); If high-risk HPV test is negative at 12 and 24 post treatment they will still require annual follow up for life (39.1%); If cytology and high-risk HPV test are negative at 12 and 24 post treatment, they will require a repeat smear in 3 Years (24.4%). In addition, more than 10% of health professionals incorrectly thought (or weren’t sure) that an HPV test can tell how long a person has had an HPV infection; an HPV test cannot be done at the same time as a Smear test; HPV testing is used to indicate if the HPV vaccine is needed; when an HPV test has been done that the results are available the same day; If an HPV test shows that a women does not have HPV her risk of cervical cancer is not low.

### HPV vaccine knowledge

Out of a maximum knowledge score of 7 (see individual questions in Table 2), the mean score achieved by the participants was 6.0 (SD 1.2) and the median score was 6 (range 0–7, IQR 5–7), with 45.9% (N = 102) achieving 100%. One individual had no answers correct.

The following questions were answered incorrectly most often: The HPV vaccine offers protection against genital warts (31.9% answered incorrectly or weren’t sure); The HPV vaccines offer protection against most cervical cancers (18.8%); The HPV vaccines are most effective if given to people who have never had sex (17.5%). In addition, more than 10% of participants incorrectly thought (or weren’t sure) that the recommended number of HPV vaccine doses was not three.

### Factors influencing level of HPV knowledge

Table 3 shows the effect of predictors on the three types of knowledge, both unadjusted (‘crude’) and adjusted for the other covariates (‘full model’). Having ever taken a smear was significantly positively associated with all three types of knowledge when entered into the model as the only predictor. However, when adjusting for the other predictors, the association with having ever taken a smear was attenuated for all knowledge types and only remained significantly associated with Triage and TOC knowledge score (where those who had ever taken a smear were more likely to have a higher knowledge score than those who had not taken a smear (OR 3.59, 95% CI 1.81–7.10, p < 0.01).

**Table 3:** Ordinal regression of predictors of knowledge.

	HPV knowledge score					
	Crude			Full model		
	OR	95% CI	p	OR	95% CI	p
Ever taken smear						
No	*Ref*			*Ref*		
Yes	2.51	1.52–4.15	<0.01	1.69	0.83–3.43	0.15
Years since HPV training						
Never	*Ref*			*Ref*		
≤1 year ago	2.85	1.44–5.63	<0.01	2.30	1.04–5.10	0.04
>1 year ago	2.27	1.25–4.14	<0.01	1.76	0.83–3.74	0.14
Current role						
Nurse	*Ref*			*Ref*		
GP	2.17	0.69–6.82	0.19	2.40	0.69–8.34	0.17
Colposcopy	1.43	0.18–11.42	0.74	1.09	0.13–8.96	0.94
Laboratory staff and other	0.64	0.35–1.16	0.14	1.08	0.54–2.15	0.83
	Triage and TOC knowledge score					
	Crude			Full model		
	OR	95% CI	p	OR	95% CI	p
Ever taken smear						
No	*Ref*			*Ref*		
Yes	5.33	3.17–8.98	<0.01	3.59	1.81–7.10	<0.01
Years since HPV training						
Never	*Ref*			*Ref*		
≤1 year ago	4.96	2.51–9.81	<0.01	2.59	1.22–5.52	0.01
>1 year ago	5.52	2.97–10.24	<0.01	2.41	1.18–4.95	0.02
Current role						
Nurse	*Ref*			*Ref*		
GP	0.93	0.32–2.77	0.90	0.89	0.27–2.89	0.84
Colposcopy	7.89	1.19–52.19	0.03	6.20	0.91–42.30	0.06
Laboratory staff and other	0.42	0.23–0.75	<0.01	0.92	0.47–1.81	0.82
	HPV vaccine knowledge score					
	Crude			Full model		
	OR	95% CI	p	OR	95% CI	p
Ever taken smear						
No	*Ref*			*Ref*		
Yes	2.55	1.55–4.20	<0.01	1.85	0.92–3.73	0.09
Years since HPV training						
Never	*Ref*			*Ref*		
≤1 year ago	2.94	1.46–5.93	<0.01	1.77	0.79–3.94	0.16
>1 year ago	1.92	1.06–3.47	0.03	0.90	0.42–1.91	0.77
Current role						
Nurse	*Ref*			*Ref*		
GP	0.47	0.16–1.36	0.16	0.46	0.14–1.52	0.21
Colposcopy	1.18	0.17–8.23	0.87	0.99	0.14–7.15	1.00
Laboratory staff and other	0.27	0.15–0.48	<0.01	0.34	0.17–0.67	<0.01

Years since HPV training was also associated with knowledge level in univariate analysis, where those who had had training (either ≤ 1 year ago or > 1 year ago) were more likely to have a higher knowledge score than those who had never had HPV training, across all types of knowledge. The association was more pronounced for those who had had more recent training (≤ 1 year ago) than for those who had training longer ago (> 1 year ago) for two out of the three domains, as expected. The association was attenuated when taking into other predictors on knowledge. However, having had HPV training ≤ 1 year ago compared to never remained significantly independently predictive of HPV knowledge score and Triage and TOC knowledge score; having had training > 1 year ago compared to never also remained significantly independently predictive of Triage and TOC knowledge score. Years since training was not predictive of HPV vaccine knowledge score after adjustment for the other predictors.

Current role was not associated with HPV knowledge score in univariate or multivariate analyses. However, current role was associated with the Triage and TOC knowledge score in univariate analysis with those who worked in colposcopy having a higher knowledge score than nurses (OR 7.89, 95% CI 1.19–52.19, p = 0.03). This association was attenuated and no longer statistically significant after adjustment for the other predictors (OR 6.20, 95% CI 0.91–42.30, p = 0.06). The number of colposcopy workers was very small (n = 4) and comprised 2 individuals who identified themselves as colposcopists and 2 who identified themselves as colposcopy nurses, so this result should be interpreted with caution. Those that were classed as laboratory staff or other were less likely to have higher Triage and TOC knowledge scores in the univariate analyses (OR 0.42, 95% CI 0.23–0.75, p<0.01, but this association disappeared after adjustment for the other predictors (OR 0.92, 95% CI 0.47–1.81, p = 0.82. The laboratory staff and other group were also more likely to have lower HPV vaccine knowledge scores than nurses in both univariate (OR 0.27, 95% CI 0.15–0.48, p < 0.01) and multivariate (OR 0.34, 95% CI 0.17–0.67, p < 0.01) models.

The effect of age on knowledge score was explored in univariate analysis as a potential predictor, but was not associated with scores for any of the three knowledge types (data not shown), so was not included in the multivariate analysis.

### Attitudes towards HPV vaccine

Of all respondents, 96.5% (N = 220) agreed or strongly agreed that they would recommend the HPV vaccine (Table 4), with a further 3.5% (N = 8) undecided (there were 2 blank responses).

**Table 4:** Attitudes and adequacy questions.

	Response N (%)					
	Strongly agree	Agree	Undecided	Disagree	Strongly disagree	Blank^1^
Would you recommend the HPV vaccine?	179 (78.5)	41 (18.0)	8 (3.5)	0	0	2
Do you think that the vaccine should be offered to men/boys as well?	168 (73.7)	47 (20.6)	12 (5.3)	1 (0.4)	0	2
Do you feel adequately informed about HPV?	30 (13.3)	114 (50.4)	52 (23.0)	27 (11.9)	3 (1.3)	4
Are you able to confidently answer HPV related questions asked by patients?	37 (16.4)	128 (56.9)	41 (18.2)	15 (6.7)	4 (1.8)	5

^1^Omitted from % calculation

In total, 94.3% (N = 215) respondents agreed or strongly agreed that men/boys should be offered the vaccine (Table 4), with 5.3% (N = 12) undecided and 0.4% (N = 1) in disagreement (there were 2 blank responses).

### Self-perceived adequacy of HPV knowledge

Only 63.7% (N = 144) respondents agreed or strongly agreed that they were adequately informed about HPV (see Table 4), 23.0% (N = 52) were undecided, while 13.2% (N = 30) disagreed or strongly disagreed (there were 4 blank responses).

Despite this, 73.3% (N = 165) respondents agreed or strongly agreed that they could confidently answer HPV related questions asked by patients (see Table 4). A further 18.2% (N = 41) were undecided and 8.5% (N = 19) disagreed or strongly disagreed (there were 5 blank responses).

Independent t-tests confirmed that the knowledge scores for general HPV knowledge, triage and test of cure knowledge and HPV vaccine knowledge were all significantly higher for those participants who felt they were adequately informed than in those who did not feel they were or who were unsure (p<0.01). The same was found for the question about feeling confident in answering patient questions.

Feeling adequately informed and feeling confident in answering patient questions were both related to having ever taken a smear, years since training, and to a much lesser extent, current role (data not shown). Therefore, the relationship between self-perceived adequacy and knowledge was explored further in multivariate analysis using binary logistic regression (Table 5).

**Table 5:** Logistic regression of the effect of knowledge on feeling adequately informed / confident in answering patient questions.

	**Feeling adequately informed**					
	Crude			Adjusted for ever taken a smear, years since training and current role		
	OR	95% CI	p	OR	95% CI	p
HPV knowledge	1.34	1.11–1.61	<0.01	1.14	0.93–1.39	0.22
Triage and TOC knowledge	1.30	1.12–1.52	<0.01	1.07	0.89–1.29	0.48
HPV vaccine knowledge	1.66	1.29–2.14	<0.01	1.65	1.21–2.24	<0.01
	**Feeling confident in answering patient questions**					
	Crude			Adjusted for ever taken a smear, years since training and current role		
	OR	95% CI	p	OR	95% CI	p
HPV knowledge	1.48	1.22–1.79	<0.01	1.22	0.98–1.52	0.08
Triage and TOC knowledge	1.62	1.36–1.94	<0.01	1.24	1.00–1.53	0.05
HPV vaccine knowledge	2.10	1.60–2.78	<0.01	2.16	1.47–3.17	<0.01

Feeling adequately informed and confident in answering patient questions were independently predicted by HPV vaccine knowledge after adjustment for the other predictors, while the associations with HPV knowledge and Triage and TOC knowledge disappeared after adjustment. Again, the number of health professionals in the colposcopy role category was very small, so these results should be interpreted with caution.

### Improving training

Suggestions for how training might be improved were provided by 36 respondents (15.7%). They wanted regular updates, more training sessions and several health professionals felt that online training and other online resources such as research, frequently asked questions and updates would be useful. A request for specific advice that should be provided to parents and simple information sheets for both primary care and patients was suggested. There were also requests to widen the provision of training beyond practice nurses to all healthcare providers, specifically including GPs, independent vaccinators and Public Health Nurses delivering the School Based Immunisation programme.

## Discussion

Although mean knowledge levels for HPV and the HPV vaccine were reasonable (with each subset of questions yielding a mean percentage correct score of between 88% and 85%, respectively), only 25.2% and 44.3% of health professionals scored 100% in each category, respectively. Research has been conducted in other countries with HPV vaccination programmes to explore health professional knowledge about HPV and the vaccination (e.g., [11, 13, 14]). These studies reveal that, consistent with our NZ results, health professional knowledge about HPV and the HPV vaccination is frequently incomplete.

An evaluation of knowledge about HPV and HPV vaccination for GP practice nurses in Leicestershire in the UK, where the vaccination has been administered through the NHS since 2008, found that although general HPV knowledge scores were quite high, there were specific gaps or weaknesses in knowledge [11] for example nearly 10% of PNs did not know that HPV causes cervical cancer and 63% believed that HPV requires treatment. Our study also revealed significant gaps in knowledge. For example, while general HPV knowledge was high, around a quarter of respondents were unaware that having sex at an early age increases the risk of getting HPV. A quarter of respondents were also unaware that HPV is so common that most sexually active people will be exposed to it in their lifetime. Research has shown that considerable stigma can be attached to a positive HPV test [15, 16] and that a lower level of education can be associated with an increase in the negative emotions and stigma that patients experience [16]. Therefore, it is vital that clinical staff are aware of the widespread nature of the virus so that they can reassure patients and reduce stigma. A third of participants did not know or were unclear that HPV does not usually need treatment. This lack of knowledge has the potential to spread misinformation and cause confusion among patients as they seek treatment that is not available. Perhaps most worryingly, 13% of respondents either believed that HPV causes HIV/AIDS or were unclear that it did not.

Other research has demonstrated a lack of complete knowledge about HPV and the HPV vaccine among health professionals. Nilsen et al explored knowledge of and attitudes to HPV infection and vaccination among public health nurses and GPS in Northern Norway in 2010, one year after the HPV vaccination was introduced for 12 year-old girls in Norway [13]. Knowledge of HPV infection, vaccine and cervical cancer was measured with 7 open-ended questions (e.g. what is the lifetime risk of a sexually active person getting HPV?). The percentage of GPs getting each question correct ranged from 26–55% while for the nurses it was 35–86%. Self-reported knowledge was considerably higher than actual knowledge. Only 47% of respondents knew that HPV infection is a necessary cause of cervical cancer.

In Malaysia there has been a school-based HPV vaccination programme since 2010. Jeyachelvi et al conducted a survey to explore HPV and HPV vaccination knowledge and attitudes in primary health clinic nurses who run the vaccination program in Kelantan, Malaysia [14]. Nurses were given 11 questions to assess their knowledge. The mean score was 5.37 with the minimum score being 0 and the maximum being 9. No question was answered correctly by more than 87.3% of respondents and the poorest question (External anogenital warts increase the risk of cancer at the same site where the warts are located. True/False) was answered correctly by only 10.6%.

Rutten et al conducted a survey exploring clinician knowledge, clinician barriers and perceived parental barriers to HPV vaccination in Rochester US [17]. They found that greater knowledge of HPV and the HPV vaccination (assessed together using an 11-item scale) was associated with higher rates of HPV vaccination initiation and completion of the 3-dose vaccination schedule, suggesting that knowledge is important in order to effectively promote HPV vaccination in addition to reducing stigmatising attitudes of clinicians identified in past research [18] and discussed in more detail below.

Knowledge about triage and test of cure in our study was lower than for HPV and HPV vaccine knowledge (mean percentage correct score of 74%) and only 9.1% of health professionals correctly answered all the answers in this section. The Leicester UK study discussed above also revealed gaps in the practice nurse knowledge about current NHS processes around HPV triage and test of cure [11]. For example, the role of HPV testing post-treatment (TOC) was misinterpreted, with only 66% acknowledging that all normal, borderline nuclear and mildly dyskaryotic samples are tested for high risk HPV post-treatment. Not all nurses felt adequately informed about HPV and a need to improve the provision of training was identified. For the triage and test of cure questions, while some questions were generally answered accurately in our study, some questions revealed uncertainty and a lack of understanding of the current guidelines. For example, fewer than half of the respondents knew that not all cervical samples showing mild cellular changes are tested for high-risk HPV (only those for women aged 30 and older are tested for HPV under the current NZ guidelines). In addition, almost a quarter of respondents did not know or were unsure that a negative HPV test means that a woman is at low risk from cervical cancer. This uncertainty is likely to be problematic when primary HPV testing is rolled out. Unlike the cell changes that are screened for currently, primary HPV screening is about identifying a woman’s risk factors. Health professionals will need to be confident in talking with women about what their positive test result means. The test of cure questions were also correctly answered by fewer than three quarters of the respondents.

In addition to knowledge, other studies have been conducted exploring health professionals’ attitudes towards the HPV vaccine. In Italy, almost all of the primary care paediatricians surveyed believed the vaccine was effective in preventing HPV related diseases in boys (92.3%) and girls (97.9%) and they also believed it was safe. Despite this only 18.4% always recommended the HPV vaccine to boys aged 11–12 compared with 77.4% who always recommended it to girls aged 11–12 [19]. In a French survey, 72.4% of general practitioners indicated that they always or often recommended the vaccine to girls aged 11–14 [20], while in a US survey, 60% of paediatricians and 59% of family doctors recommended the vaccine to girls aged 11–12 compared with 52% and 41% who recommended it to boys aged 11–12 [21]. By contrast in our survey, the vast majority of health professionals indicated that they would recommend the vaccine and they also favoured vaccinating boys and men, with only one individual indicating they would not recommend vaccinating boys and men. This is particularly reassuring since NZ made the vaccine available to boys from January 2017.

### The public and HPV

The public is generally not well informed about HPV and its health sequelae, even in countries with well-established vaccine and screening programmes, and the role of health professionals is vital in mitigating this lack of knowledge [22, 23]. For example, in a survey of 200 NZ university health science students (mean age 19.8 years), 50.8% were both unaware of the sexual transmission of HPV and unwilling to accept a free HPV vaccine, highlighting the need for education in this age group [24]. In the UK, Sherman and Nailer found that only half of parents of teenage boys in their survey had heard of HPV and the HPV vaccine, despite the vaccine having been available to girls in the UK since 2008 [25]. The HPV vaccination programme in the UK is school-based and Boyce and Holmes identified that the school nurse played a vital role in reducing health inequalities associated with vaccine uptake [26]. In another survey, of young adults exploring psychological traits and vaccine uptake in the US, Scherer et al suggested that women who receive the HPV vaccine may do so based on informational evidence and that for both males and females, information about the vaccine “should be communicated in a way that highlights the risks associated with HPV and reduces uncertainty about the HPV vaccine” [27].

HPV-related knowledge or lack thereof can also impact cervical screening engagement. A survey of adult women in Kenya from HIV-1-discordant couples, found that those women who had never attended screening reported not knowing what a Pap smear was or why they needed one. After adjusting for age, both education and knowledge of HPV were associated with ever having a smear test [28]. In a survey of women who underwent first time treatment for high grade cervical intraepithelial neoplasia (CIN) in Sweden, knowledge about HPV, CIN and cervical cancer predicted their understanding of their personal risk of cervical cancer [29] and Barnoy et al have previously identified that lower unrealistic optimism about risk was associated with intention to undergo screening tests [30]. Crucially, more than two thirds of the Swedish women surveyed stated they would like “to receive more information about HPV, cervical cancer and its prevention from health professionals (midwives, gynaecologists, primary care physicians)” [29]. Furthermore, there is considerable stigma associated with a diagnosis of HPV [15, 16]. For example, in a qualitative study, McCaffrey et al., found that HPV positive women [15] reported levels of stigma and anxiety suggesting that “testing positive for HPV was associated with adverse social and psychological consequences that were beyond those experienced by an abnormal smear alone” (p173). Daley et al., found that younger age and less education were associated with more negative emotions (e.g., anger, shock and worry) and stigma beliefs (e.g., feeling ashamed, guilty and unclean) in HPV positive women [16]. In addition, a survey of Hong Kong Chinese healthcare providers exploring levels of knowledge about HPV and attitudes revealed that more knowledge about HPV predicted less stigmatising attitudes from healthcare providers [18].

The findings above underscore the need for health professionals to be well informed about HPV, the vaccine and screening programme.

### Health professional education needed

Our results suggest that education about HPV and particularly the use of HPV testing in the screening programme and test of cure process is urgently needed to address some worrying gaps in knowledge. This is especially important since further changes to the screening programme are due to be implemented, with draft primary HPV screening guidelines recently out for consultation [31]. As other countries also start to roll out primary HPV screening, the success of primary screening engagement in NZ and the rest of the world may well rest upon the level of knowledge of those health professionals responsible for implementing it.

The need for education indicated by the knowledge scores was further reinforced by the fact that over a third of respondents did not agree that they felt adequately informed about HPV and that being adequately informed and feeling confident in responding to patients’ questions were both associated with knowledge. Suggestions for training were proposed by some of the respondents. One promising suggestion, which was also proposed by UK practice nurses [11], was for online training. This would provide a low-cost way to update changes to the vaccination and/or screening programmes and guidelines in a format that would be easily accessible to many staff whilst requiring relatively little time commitment to complete. HPV vaccination online training was developed by the Immunisation Advisory Committee (IMAC) and was released in August 2017 [32]. This may address some of the knowledge issues associated with vaccination identified in this study, but additional online training regarding screening and test of cure is needed.

### Limitations

There are several limitations to our study. Firstly, due to the opportunistic recruitment approach, involving self-selection of study participants, the response rate is unknown. As a consequence, we were not able to examine whether, and to what extent, bias due to non-response (or participation bias) has occurred, as we were unable to assess the level of HPV knowledge in non-responders. Also, as is common for most questionnaire surveys, we cannot exclude social desirability bias (the tendency of survey respondents to answer questions in a manner that will be viewed favourably by others), but believe that this type of bias is less likely to be a significant factor for health professionals. Secondly, the sample is not evenly distributed across the categories of health professionals, with significantly more nurses having completed the survey than GPs, colposcopists or laboratory and other staff. Since there are currently no official data available on the cervical screening workforce in New Zealand, we are unable to indicate how representative our sample is of that wider group. Thirdly, some of the questions, such as whether participants felt they could confidently answer HPV related questions asked by patients, are less relevant to laboratory staff who are less likely to interact with patients. Lastly, as with all such surveys, by providing questions with true/false/don’t know response options, the questions themselves might act as a prompt enabling educated guesses rather than measuring knowledge directly and thus might potentially overestimate knowledge.

### Concluding comments

Our survey is the first to be conducted in NZ that explores health professional knowledge and understanding about HPV, the vaccine and the role of HPV testing in the cervical screening programme and it contributes to the international picture about HPV knowledge that is emerging. It is evident from our findings and those from other countries, that more education is required to ensure that misinformation, the stigma associated with the sexually transmitted nature of HPV and widening inequalities do not inadvertently result from interactions between health professionals and the public.
